# Relationship between psychopathology and binge size in binge eating spectrum disorders

**DOI:** 10.47626/2237-6089-2023-0644

**Published:** 2024-11-26

**Authors:** Carla L. Mourilhe Silva, Gloria Valeria da Veiga, Carlos Eduardo de Moraes, Ronir Raggio Luiz, Phillipa Hay, Jose Carlos Appolinario

**Affiliations:** 1 Universidade Federal do Rio de Janeiro Instituto de Psiquiatria Grupo de Obesidade e Transtornos Alimentares Rio de Janeiro RJ Brazil Grupo de Obesidade e Transtornos Alimentares, Serviço Especializado, Instituto de Psiquiatria, Universidade Federal do Rio de Janeiro (UFRJ), Rio de Janeiro, RJ, Brazil.; 2 UFRJ Instituto de Nutrição Josué de Castro Departamento de Nutrição Social e Aplicada Rio de Janeiro RJ Brazil Departamento de Nutrição Social e Aplicada, Instituto de Nutrição Josué de Castro, UFRJ, Rio de Janeiro, RJ, Brazil.; 3 UFRJ Instituto de Estudos em Saúde Pública Rio de Janeiro RJ Brazil Instituto de Estudos em Saúde Pública, UFRJ, Rio de Janeiro, RJ, Brazil.; 4 Western Sydney University School of Medicine Translational Health Research Institute Sydney NSW Australia Translational Health Research Institute, School of Medicine, Western Sydney University, Sydney, NSW, Australia.

**Keywords:** Binge eating size, psychopathology, eating disorders, binge eating disorder, bulimia nervosa

## Abstract

**Objective:**

Food intake during binge eating episodes (BEE) has been found to be associated with symptoms of depression and anxiety in individuals with eating disorders (EDs). The objective of this study was to evaluate the association between caloric intake during BEE and psychopathology in individuals with binge eating spectrum disorders (BSD).

**Methods:**

One-hundred and fourteen outpatients diagnosed with bulimia nervosa (BN) and binge eating disorder (BED) were sequentially assessed. The Mini International Neuropsychiatric Interview Plus (MINI PLUS) was used to assess psychiatric diagnoses. Validated self-report instruments were used to assess general and eating-related psychopathology. The Dietpro Clinical Program^®^ was used for assessment of calorie consumption during BEE. Data were analyzed with independent Student's t tests, effect size (Cohen's *d*), and Pearson's correlation coefficients.

**Results:**

Participants with BSD comorbid with a depressive disorder consumed significantly more calories during BEE than those without depression. Furthermore, participants with BSD and higher levels of impulsivity had higher caloric intake during episodes. Specifically regarding BN, participants with greater disease severity consumed more calories during episodes than those with lesser severity.

**Conclusion:**

Overall, depression and high impulsivity were associated with higher caloric intake during BEE in individuals with BSD. For those with BN, disease severity was associated with greater calorie consumption during episodes. Our results support the relevance of early identification of psychiatric comorbidities and implementation of strategies to control mood and impulsivity, aiming for better prognosis in the treatment of BSD.

## Introduction

Binge eating is a cardinal symptom present in binge eating spectrum disorders (BSD) such as bulimia nervosa (BN) and binge eating disorder (BED).^1^ Since early reports by Stunkard, two main features of binge eating have been identified: excessive consumption of food and an experience of loss of control over eating.^2^ However, while the loss of control over eating criterion is currently accepted as an essential component of binge eating, the validity of the binge eating size criterion is less clear.^3-6^

Some authors suggest that binge eating size could be a marker of severity along with other aspects of this phenomenon.^7^ Food intake during binge eating episodes (BEE) is associated with symptoms of depression and anxiety in individuals with eating disorders (EDs).^6,8-10^ Studies have suggested that individuals may use binge eating as a strategy to distract themselves from their negative emotions, such as sadness, anxiety, or anger. This relationship may be especially relevant in individuals with EDs, who often have emotional and affective regulation difficulties.^11,12^

Eating disorders have been associated with other mental health conditions. For instance, depression has been frequently diagnosed in individuals with EDs. In addition, studies have suggested a bidirectional relationship between binge eating and depression, with each of these conditions influencing and aggravating the other. Similarly, anxiety may precede binge episodes and increase the likelihood of recurrent BEE.^6,8-10^

Moreover, impulsivity has been associated with binge eating symptoms and ED diagnosis.^13,14^

A systematic review of 43 studies assessing binge eating characteristics found a positive correlation between depressive mood and caloric intake during BEE. It is noteworthy that the meta-analysis considered only studies that assessed objective BEE (consumption of a large amount of food associated with the feeling of loss of control over eating) due to the lack of a consistent number of studies that considered the definition of subjective BEE (the feeling of loss of control over eating associated with the consumption of small or moderate amounts of food). This review identified some limitations of the current literature about binge eating size: (1) few studies correlated caloric intake with BEE and/or other psychopathological measures; (2) heterogeneity of methodologies, which limited the comparisons among the studies; and (3) a decline in the number of publications on the subject in recent years.^15^

Considering these gaps in the literature, there is a need for better understanding of the relationship between binge eating size and psychopathology, especially symptoms of depression, anxiety, and impulsivity, which is of paramount importance for progress in this area of ED research. This relationship may provide valuable information for understanding the underlying mechanisms and developing more effective therapeutic interventions. Hence, the present study assessed the potential associations between caloric intake during objective BEE and general and eating-related psychopathology in patients with BSD.

## Methods

### Study design and setting

This is a cross-sectional study carried out in an outpatient ED unit. The study was conducted between 2017 and 2019 with outpatients diagnosed with BSD seeking treatment for their conditions at the Grupo de Obesidade e Transtornos Alimentares (GOTA), Instituto de Psiquiatria (IPUB), Universidade Federal do Rio de Janeiro (UFRJ), Brazil.

### Participants

Participants were sequentially invited to participate in this study. Participants were adolescents (over 16 years) or adults meeting the criteria for BN, BED, and their subthreshold forms according to the Diagnostic and Statistical Manual of Mental Disorders, 5th edition (DSM-5). Children and pregnant or lactating women were excluded.

### Ethical considerations

The study protocol was approved by the IPUB/UFRJ Institutional Ethics Committee (CAAE 65842817.9.0000.5263). Participants signed informed consent before taking part in any study procedures.

### Procedures

In the baseline assessment, participants were diagnosed by structured interview. Next, sociodemographic and clinical information, including age, sex, ethnicity, marital status, schooling, occupation, age at disease onset, and time since onset were collected. Anthropometric variables were measured next and then the nutritional evaluation was carried out, including an investigation of food consumption during a binge eating episode. General and eating-related psychopathologies were investigated using validated psychiatric assessment instruments. Data were collected before treatment was started, as part of the service's initial screening.

### Measures

#### Psychiatric assessment

**Diagnosis of eating disorders and psychiatric comorbidities.** Diagnoses of BN, BED, and their subthreshold forms were confirmed with the Structured Clinical Interview for DSM-IV (SCID-I/P) adapted for DSM-5.^1^ Comorbid psychiatric conditions were assessed using the mood and anxiety schedule of the Mini International Neuropsychiatric Interview Plus (MINI PLUS), also administered by trained psychiatrists.^16,17^ Interviews were conducted by trained psychiatrists specialized in the area of EDs.

**Eating disorder severity.** In the initial assessment, the clinician measured ED severity using the Clinical Global Impression (CGI). Scores ranged from 0 to 7, where higher values mean greater severity (0 = unvalued/1 = without symptoms/7 = extremely sick). Severity levels were classified as follows: a) mild (scores of 1-3); b) moderate (4); c) severe (scores of 5-7).^18^

**General and eating-related psychopathology.** The severity of depressive symptoms was assessed using the 21-item Beck Depression Inventory-II (BDI-II), a self-report questionnaire in which respondents describe how they have been feeling in the previous week.^19^ In general, the internal consistency of BDI-II has been described as good to excellent.^20^ The severity of depressive symptoms was classified according to the following score brackets: a) without symptoms (0-9 points); b) mild (10-18); c) moderate (19-29); or d) severe (30-63). The internal consistency of the Portuguese version of BDI-II was 0.88 for subjects with depression.^21^

**Anxiety.** Anxiety symptoms were evaluated using the 20-item State-Trait Anxiety Inventory (STAI). Anxiety is assessed as state (STAI-S), a transient reaction of the organism to a certain situation or moment, and as trait (STAI-T), individual differences related to how a given individual deals with greater or lesser anxiety throughout life.^22^ Studies carried out in Brazil have indicated high internal consistency for both sub-scales.^23^ The score for each part of the instrument varies from 20 to 80 points. The severity of anxiety symptoms was classified as: a) low (0-32 points); b) moderate (33-49); or c) high (greater than or equal to 50).^21^

**Impulsivity.** The Barratt Impulsiveness Scale (BIS-11), a 30-item self-report instrument, was used to assess impulsivity. The model proposes that impulsivity is a multidimensional construct that encompasses three factors/components expressed in the following subscales: (1) motor (ability to contain the act); (2) attentional (quick decision making); and (3) lack of planning (orientation to the present).^24,25^ The instrument showed good internal consistency for the total score.^26^ Scores range from 30 to 120 points and higher scores are associated with greater impulsivity. The instrument classifies individuals according to the following levels of severity: a) very controlled (less than 52 points); b) normal impulsivity (52-71); or c) highly impulsive (greater than 72).^24^

**Bulimia nervosa.** In participants with BN, bulimic behaviors were assessed by the Bulimic Investigatory Test, Edinburgh (BITE). This self-report instrument is composed of two sub-scales, one focusing on the symptoms and the other on their severity (frequency of binge eating and compensatory behaviors). The symptoms subscale classifies individuals into the following groups: a) low (normal limits) (< 10 points); b) medium (unusual eating pattern) (10 to 19); or c) high (compulsive eating behavior) (≥ 20). The severity subscale is divided into three categories: a) low (< 5 points); b) moderate (5 to 9); or c) high (≥ 10).^27,28^

**Binge eating disorder.** In participants with BED, the severity of binge eating was assessed using the Binge Eating Scale (BES), a 16-item self-report instrument that considers the frequency of episodes, the amount of food eaten, and the degree of emotion involved in BEE. BES scores are classified into the following levels of severity: a) < 17, no binge eating; b) 18 to 26, moderate binge eating; or c) ≥ 27, severe binge eating.^29,30^

#### Energy intake (kcal)

For the assessment of energy intake, participants were instructed to recall a representative binge eating episode, defined according to DSM-5 (objectively large amount of food plus loss of control), that occurred during the last month.^31^ In the nutritional interview, after the description of the food items consumed during the episode, the quantities reported were reviewed by a registered dietitian (CM), using images of portion sizes for greater accuracy of the description. Furthermore, the foods consumed were characterized according to their brands and cooking methods to provide a detailed description of the food consumed during the episode. Next, we checked if any other products were added to the preparations (e.g., sugar/sweeteners or condiments) and if any drinks or other foods consumed had not been mentioned.^32^ Finally, all the data regarding the foods and beverages consumed during the BEE were input to the Dietpro Clinical Program^®^, version 5.8.^33^

#### Anthropometric measurements

Participants’ weight and height were measured using a WELMY^®^ electronic beam scale and a TONELLI^®^ wall stadiometer. These measurements were performed with the participant barefoot and wearing light clothing. The weight and height measurements were used to calculate body mass index (BMI) (kg/m²).

### Statistical analysis

First, the participants’ characteristics were presented descriptively, with continuous variables expressed using means and standard deviations (SD) and categorical variables using absolute and relative frequencies. The median, minimum, maximum, and interquartile range of the dependent variable (caloric intake during the episode) were also presented. Data were inspected for normality.

In the next step, caloric intake during the episode was compared with psychiatric diagnoses and measures of general and eating related psychopathology. The severity of psychopathological symptoms was categorized according to the literature, as described previously in "Measures." Associations with binge eating size were analyzed based on the high severity categories. The independent Student's *t* test and effect size (Cohen's *d*) were used to compare two means. The effect size was considered small when Cohen's *d* was less than 0.2, medium if 0.4, and large when 0.6.^34^ Categorical variables were compared using the chi-square test. Differences between groups were considered statistically significant when p ≤ 0.05. The degree of association between continuous variables was analyzed using Pearson's correlation coefficients. Correlations were considered statistically significant when moderate or strong with p ≤ 0.05. Statistical analyses were performed using the Statistical Package for the Social Sciences, SPSS/21.0.

## Results

**Figure 1:**
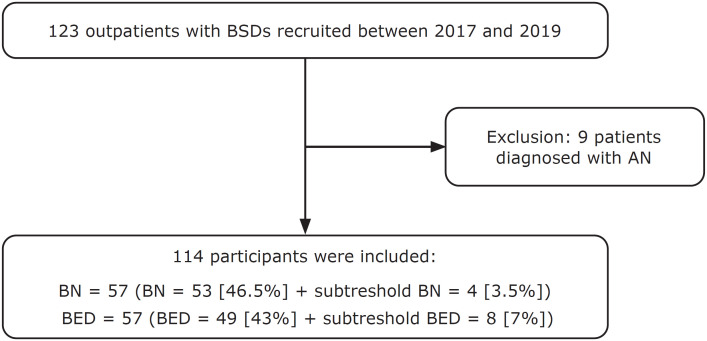
Recruitment flow chart. AN = anorexia nervosa; BED = binge eating disorder; BN = bulimia nervosa; BSDs = binge eating spectrum disorder.

The study included 114 participants with BSD. Fifty percent had a diagnosis of BN (n = 57) and 50% (n = 57) had BED (Figure 1). The mean age of participants with BSD was 30.3 (SD = 9.9) years, significantly higher in participants with BED 36.4 (SD = 13.1) than in those with BN 33.4 (SD = 12) (p = 0.006). Eighty-eight percent of the participants were women. BMI was significantly lower in participants with BN than in those with BED (28.5 [SD = 7.5] vs. 40.6 [SD = 8.9] kg/m², p = 0.000) and age of disease onset was younger in the BN group (16.6 [SD = 6.1] vs. 21.2 [SD = 10.5] years, p = 0.005). There were no differences in time since onset or disease severity between groups (Table 1). It is noteworthy that patients with BN tended to be considered more severe according to the CGI (4.5 [SD = 1.2] vs. 4.1 [SD = 0.9] years, p = 0.054) and had higher impulsivity scores (75.2 [SD = 12.9] vs. 70.2 [SD = 12.6] years, p = 0.049) (Supplementary Table S1. Overall, participants with BSD consumed a mean of 2,106.6 (SD = 1,327.3) Kcal during BEE. We did not observe a statistically significant difference in caloric intake between participants with BN (2263.8 [SD = 1353.4] Kcal) and BED (1949.4 [SD = 1293.5] Kcal, p = 0.207) (Table 2).

**Table 1:** Sociodemographic and clinical characteristics of the participants with BSD (n = 114)

Sociodemographic and clinical characteristics		Mean (SD)
Age		33.4 (12.0)
BMI (Kg/m²)		34.6 (10.2)
Age at disease onset (years)		18.9 (8.9)
Time since onset (months)		171.9 (128.0)
		**n (%)**
Self-reported gender		
	Male	14 (12.3)
	Female	100 (87.7)
Self-reported race		
	White	72 (63.2)
	Mixed	22 (19.3)
	Black	20 (17.5)
Marital status		
	Single	81 (71.1)
	Married	21 (18.4)
	Separated/widowed	12 (10.5)
Education		
	Elementary	17 (14.9)
	High school	13 (11.4)
	Undergraduate	31 (27.2)
	Graduated	40 (35.1)
	Postgraduate	13 (11.4)
Work status		
	Never worked	21 (18.4)
	Employee	51 (44.7)
	Unemployed	27 (23.7)
	Licensed/retired	15 (13.2)
Eating disorder diagnoses		
	BN	57 (50.0)
	BED	57 (50.0)

BED = binge eating disorder; BMI = body mass index; BN = bulimia nervosa; BSD = binge eating spectrum disorders; M = mean; SD = standard deviation.

**Table 2:** Caloric intake during BEE in participants by ED status

	BSDs (n = 114)			BN (n = 57)			BED (n = 57)			
	Mean (SD)	Median (Min-Max)	IQR	Mean (SD)	Median (Min-Max)	IQR	Mean (SD)	Median (Min-Max)	IQR	p-value†
Caloric intake (Kcal)	2106.6 (1327.3)	1859.8 (339.1-8944.3)	(1143.3/2600.2)	2263.8 (1,353.4)	2,075.6 (339.1-5850.8)	(1243.3/2789.1)	1949.4 (1293.5)	1665.2 (652.1-8944.3)	(1100.3/2322.9)	0.207

BED = binge eating disorders; BEE = binge eating episodes; BN = bulimia nervosa; BSDs = binge eating spectrum disorder; ED = eating disorder; IQR = interquartile range; Kcal = calories; Max = maximum; Min = minimum; SD = standard deviation.

† *t* test.

Depressive (68% [n = 68]) and anxiety disorders (60.2% [n = 62]) were the most prevalent psychiatric diagnoses associated with BSD. However, there were no statistically significant differences between participants with BN and BED. Conversely, individuals with a BSD and a depressive disorder had a higher caloric intake during BEE than those without a diagnosis of depression (2323.6 [SD = 1490.8] vs. 1770 [SD = 965.4], p = 0.02) Kcal with a moderate effect size in this group (0.41) as well as for those with BED and depression (0.48). Conversely, in the group with BN and depression, the effect size was small (0.35). Regarding patients with BN, those with comorbid anxiety disorders showed a trend towards a higher caloric intake during the episode (2612.3 [SD = 1478.1] vs. 1929.9 [SD = 1143.6], p = 0.060 Kcal with a moderate effect size (0.51) (Table 3).

**Table 3:** Associations between caloric intake during BEE and psychiatric comorbidities, general, and eating-related psychopathology according to ED diagnosis

			Total			BN			BED		
			n	Mean (SD)	p-value (Cohen's d)	n	Mean (SD)	p-value (Cohen's d)	n	Mean (SD)	p-value (Cohen's d)
Associated psychiatric comorbidities (MINI PLUS)											
	Depressive disorders										
		No depression	35	1770.0 (965.4)	**p = 0.025 (0.41)**	18	1981.3 (1167.1)	p = 0.234 **(0.35)**	17	1546.3 (655.7)	p = 0.117 **(0.48)**
		Depression	68	2323.6 (1490.8)		36	2453.9 (1443.8)		32	2177.0 (1551.8)	
	Anxiety disorders										
		No anxiety	41	2026.7 (1534.0)	p = 0.511 (0.13)	25	1929.9 (1143.6)	p = 0.062 **(0.51)**	16	2177.9 (2035.4)	p = 0.546 (0.24)
		Anxiety	62	2207.5 (1233.1)		29	2612.3 (1478.1)		33	1851.7 (841.33)	
Psychopathological symptoms (specific symptom scales)											
	Symptoms of depression (BDI)										
		No severe depression	66	2075.5 (1413.1)	p = 0.745 (0.07)	34	2228.8 (1327.6)	p = 0.551 (0.17)	32	1912.5 (1502.6)	p = 0.952 (0.02)
		Severe depression	38	2166.8 (1297.2)		18	2474.8 (1548.6)		20	1889.6 (908.1)	
	Severity of state anxiety (STAI-S)										
		No high anxiety	87	2055.2 (1358.5)	p = 0.238 **(0.32)**	46	2193.4 (1325.3)	p = 0.108 **(0.66)**	41	1900.1 (1394.7)	p = 0.795 (0.10)
		High anxiety	16	2496.7 (1416.0)		7	3096.9 (1614.9)		9	2029.8 (1115.0)	
	Severity of trait anxiety (STAI-T)										
		No high anxiety	51	2110.6 (1273.4)	p = 0.923 (0.02)	25	2391.7 (1571.9)	p = 0.699 (0.11)	26	1840.3 (846.3)	p = 0.652 (0.13)
		High anxiety	52	2136.7 (1470.8)		28	2242.3 (1217.9)		24	2013.5 (1739.5)	
	Impulsiveness (BIS-11)										
		No high impulsivity	48	1859.8 (1049.7)	**p = 0.056 (0.38)**	20	2198.4 (1257.9)	p = 0.669 (0.12)	28	1618.1 (811.7)	**p = 0.064 (0.58)**
		High impulsivity	57	2370.5 (1559.2)		34	2366.1 (1450.7)		23	2376.9 (1741.2)	
Eating psychopathology (specific symptom scales)											
	Global clinical severity (CGI-S)										
		Not severely ill	80	1971.7 (1117.8)	p = 0.358 (0.24)	38	2130.7 (1233.3)	p = 0.393 (0.27)	42	1827.8 (995.2)	p = 0.582 (0.26)
		Severely ill	19	2255.6 (1533.1)		14	2499.2 (1696.1)		5	1573.4 (652.5)	
	Symptoms of BN (BITE-S)										
		Not high	NA			5	1276.3 (± 795.5)	**p = 0.092 (0.82)**	NA		
		High				39	2401.4 (±1420.0)				
	Severity of BN (BITE-G)										
		Not high	NA			15	1709.3(± 907.9)	**p = 0.052 (0.64)**	NA		
		High				29	2574.3 (± 1534.3)				
	Severity of binge eating (BES)										
		No severe binge eating	NA			NA			12	1586.8 (653.9)	p = 0.315 **(0.35)**
		Severe binge eating							30	1882.6 (914.1)	

BDI = Beck Depression Inventory (BDI > 30 points – high severity); BED = binge eating disorder; BEE = binge eating episodes; BES = Binge Eating Scale (> 27 points, high severity); BIS-11 = Barrat Impulsivity Scale (> 72 points, high severity); BITE = Bulimic Investigatory Test, Edinburgh (Symptoms subscale > 20 points, high level of symptoms; Severity Subscale > 10 points, high level of severity); BITE-G = BITE, Severity scale; BITE-S = BITE, Symptoms scale; BN = bulimia nervosa; CGI-S = Global Clinical Impression Scale (> 5 points, high severity); ED = eating disorder STAI-S = State-Trait Anxiety Inventory - State; STAI-T State-Trait Anxiety Inventory - Trait (STAI > 50 points – High severity); MINI PLUS = Mini International Neuropsychiatric Interview Plus; NA = not applicable; SD = standard deviation.

Independent test *t*. Values in bold type indicate significant differences between groups or a substantial effect size.

Participants with BSD and a high level of impulsivity had a higher caloric intake during BEE compared to those without high impulsivity (2370.5 [SD = 1559.2] vs. 1859.8 [SD = 1049.7] Kcal, p = 0.05] with a small effect size (0.38). In patients with BED, there was a trend towards a higher caloric intake during the episode in the presence of high impulsivity (2376.9 [SD = 1741.2] vs. 1618.1 [SD = 811.7] Kcal, p = 0.060 with a moderate effect size (0.58). Symptoms of depression (BDI-II) and anxiety (STAI) and disease severity (CGI) showed no associations with caloric intake during the episode. However, when comorbid anxiety was assessed using the STAI-S, it had a strong effect size on binge eating size (0.66) (Table 3).

Among participants with BN, there was a trend towards a greater caloric consumption among those with high levels of bulimic symptoms (2,401.4 [SD = 1,420] vs. 1276.3 [SD = 795.5) kcal, p = 0.09) with a large effect size (0.82). Moreover, individuals with high disease severity consumed significantly more calories during BEE (2,574.3 [SD = 1534.3] vs. 1709.3 [SD = 907.9] Kcal, p = 0.05) with a large effect size (0.64). Conversely, among participants with BED, there was no statistically significant difference in the caloric consumption during binge eating related to disease severity (Table 3).

Analysis of Pearson's correlation coefficients to assess whether caloric intake during the BEE correlated with the symptoms of general and eating psychopathology in individuals with ED did not show an association (Supplementary Table S2). Only a moderate correlation was detected between caloric intake during the episode and severity of BN symptoms (BITE-Symptoms) (r = 0.438, p = 0.03) (Supplementary Table S3). Among participants with BED, no correlation was observed between caloric intake during BEE and the other variables (Supplementary Table S4).

## Discussion

The current study aimed to investigate the associations between caloric intake during BEE and general and eating-related psychopathology in outpatients with BSD. To the best of our knowledge, this study was the first to assess associations between calories consumed during BEE and categorical diagnoses of psychiatric comorbidities. Participants with BSD comorbid with depressive disorders consumed a significantly greater number of calories during BEE and, among participants with BN, those diagnosed with anxiety disorder had a higher caloric intake during the episode. In addition, high levels of impulsivity were associated with higher caloric intake in people with BSD. Furthermore, the severity of BN was positively associated with the caloric intake during binge eating.

These findings were partly consistent with a recent systematic review, which found a positive association between binge eating size and the severity of depressive symptoms.^15^ Although we did not observe associations with depressive symptoms dimensionally, we found an association between the diagnosis of depression and a higher caloric intake during the episode in individuals with BSD. Conversely, the relationship between anxiety and binge eating size is still controversial.^15^ We observed a trend to an association between state anxiety and caloric intake during the episode in participants with BN with a moderate effect size. Latner et al.^6^ found that although objective and subjective BEE were similarly related to general and eating psychopathology, mood-related psychopathology was exclusively associated with the consumption of large amounts of food during episodes. Similarly, in the present study, individuals with BSD and depressive disorders consumed a greater amount of food during episodes. On the other hand, Brownstone et al.^35^ found that individuals with BN had similar levels of ED symptoms and negative affect (symptoms of depression and anxiety) regardless of the amount ingested during the episode.

There are several possible explanations for these findings. First, an increase in caloric intake may be a consequence of ED symptomatology. Therefore, those with this disease use binge eating to relieve or escape from symptoms of depression and anxiety. Second, some features of EDs, such as distress and loss of control over eating, increased internalization of symptoms and then depression increases food intake by increasing appetite. Finally, presence of BEE and elevated symptoms of depression and anxiety, synergistically influence food intake.^10^

We found a positive association between high impulsivity traits and the amount of food eaten during BEE in participants with BSD. This is consistent with previous studies that showed an association between impulsivity and EDs.^13,14,36^ In a systematic review of 12 studies assessing impulsivity in adults with EDs using self-report instruments, Waxman^13^ found that high impulsivity was mainly associated with binge eating and purgative behaviors. Another recent systematic review of studies with participants with BED investigated the relationship between impulsivity and food consumption. Some of the included studies showed a positive correlation between impulsivity and the food consumed.^36^ One potential explanation for these findings is that impulsivity is linked to a deficient inhibitory process, so highly impulsive individuals might therefore make hasty decisions about food.^14^

Forney et al.^7^ carried out a study with 243 women with BN and purgative disorder to assess the validity of the DSM-5 size criterion, regardless of the effect of loss of control. Their results suggested that binge eating size can provide information about the severity of the ED due to its relationship with disinhibition, depressive symptoms, and anxiety, which is not explained by presence or severity of loss of control alone. The frequency of purging and depressive symptoms was higher in the presence of larger BEE. Although this study did not include individuals with BED, some of its results are similar to what we have found.

We found an association between BN severity, assessed by the BITE severity subscale, and greater caloric intake during the episode. The BITE severity subscale quantifies the severity of BN using the frequency of BEE and the number of compensatory behaviors.^28^ Thus, our findings may suggest an association between the frequency of binge eating and use of compensatory behaviors and the size of the episode. Similarly, Latner et al.^6^ found that eating large amounts of food during BEE was related to the frequency of self-induced vomiting. These findings reinforce the importance of characterizing the binge eating size, which may be valuable as a criterion to specify severity.^7^

It is noteworthy that assessment of the caloric intake during BEE yields an imperfect measure of the amount of food eaten. This can introduce a bias in studies investigating the relationship between binge eating size and psychopathology.^37^ Additional limitations of the present study include the lack of a sample size calculation and the use of self-report scales for assessment of psychopathology. However, these instruments have been validated in the Brazilian setting and showed satisfactory internal consistency. The small sample size weakened the analysis of the data. Thus, we discussed statistical significance and effect sizes. Due to this limitation, we were also unable to perform regression analysis and multiple control tests.

As this is an exploratory study, we have not analyzed possible confounders that may have influenced food intake, such as physical activity and eating restriction. This study considered BEE defined according to DSM-5, which may end up excluding subjective BEE. However, the last review on this topic also only considered objective episodes in its meta-analysis and likewise verified the correlation of binge eating size with depressive symptoms.^15^ Nevertheless, this study’ strengths were: (1) the use of standardized interviews conducted by psychiatrists specialized in EDs for diagnosis of psychiatric comorbidities; (2) the assessment of BEE by a specialized and trained dietitian; and (3) assessment of the participants included in the study before they had started ED treatment.

Our findings are relevant in clinical settings since psychopathological variables such as depression and impulsivity, as well as the severity and frequency of bulimic behaviors in BN may be related to higher caloric intake during BEE, impacting the nutritional status and clinical course of individuals with BSD. Therefore, early identification of psychiatric comorbidities and implementation of strategies to reduce impulsive behaviors may represent potential approaches to treating and preventing BSD.

Our results support the relevance of binge eating size in combination with loss of control as currently described in the DSM-5 criteria. Longitudinal studies are needed to provide a better understanding of the similarities and differences between those who exhibit objective and subjective BEE in terms of diet and psychopathology, as well as to understanding the relationship between food consumption during the episode (i.e., food portions and caloric intake), psychopathology, and loss of control eating. Although the ICD-11 omits the requirement of the size criterion for identification of BEE, our findings support the clinical utility and further study of this feature as a diagnostic specifier.

## Conclusion

The presence of a depressive disorder and high impulsivity was associated with a higher caloric intake during BEE in individuals with BSD. In addition, among those with BN, disease severity may play an additional role in the caloric consumption during the episode.
