# Supplementary material for: Relationship between psychopathology and binge size in binge eating spectrum disorders
Source: Trends Psychiatry Psychother. 2024 Nov 26;46:e20230644. doi: 10.47626/2237-6089-2023-0644 (PMC11790107; doi:10.47626/2237-6089-2023-0644)
Supplement: Supplementary file 1 [file 2238-0019-trends-46-e20230644-suppl01.pdf]

http://doi.org/10.47626/2237-6089-2023-0644

Supplementary Table S1- Severity of symptoms of general and eating related psychopathology according to ED state

	Total		Diagnosis				
			BN		BED		
	n	Mean (SD)	n	Mean (SD)	n	Mean (SD)	p-value
Symptoms of depression (BDI)	105	26.3 (± 12.4)	52	25.5 (± 11.0)	53	27.2 (± 13.7)	p = 0.490
Severity anxiety state (STAI-S)	103	42.9 (± 8.9)	53	42.6 (± 9.2)	50	43.3 (± 8.8)	p = 0.687
Severity anxiety trace (STAI-T)	103	49.0 (± 9.1)	53	49.5 (± 9.2)	50	48.6 (± 9.0)	p = 0.622
Impulsiveness (BIS-11)	105	72.8 (± 12.9)	54	75.2 (± 12.9)	51	70.2 (± 12.6)	**p = 0.049**
Symptoms of BN (BITE-S)			44	24.7 (± 3.8)			
Severity of BN (BITE-G)			44	12.9 (± 6.8)			
Severity of binge eating (BES)					42	31.9 (± 8.5)	
Clinical global impression (CGI-S)	99	4.3 (± 1.1)	52	4.5 (± 1.2)	47	4.1 (± 0.9)	**p = 0.054**

BDI = Beck Depression Inventory; BIS-11 = Barrat Impulsivity Scale; BITE = Bulimic Investigatory Test, Edinburgh (S = Symptoms subscale, G = Severity Subscale); BES – Binge Eating Scale; BN = bulimia nervosa; CGI-S = Global Clinical Impression Scale; ED = eating disorder; STAI-S = State Anxiety Inventory; STAI-T = Trait Anxiety Inventory.

Supplementary Table S2 - Pearson's correlation coefficients and level of significance between caloric intake during BEE and levels of general psychopathology in eating disorders

Diagnosis	Caloric intake	BDI	STAI-S	STAI-T	BIS-11
Caloric intake					
ρ	1	-0.028	0.028	-0.005	0.160
p-value		0.778	0.776	0.963	0.102
n	114	105	103	103	105

BDI = Beck Depression Inventory; BEE = binge eating episodes; BIS-11 = Barrat Impulsivity Scale; STAI-S = State Anxiety Inventory; STAI-T = Trait Anxiety Inventory.

Supplementary Table S3- Pearson's correlation coefficients and level of significance between caloric intake during BEE and levels of general and eating related psychopathology in BN

Diagnosis	Caloric intake	BITE-S	BITE-G	BITE Total	BDI	STAI-S	STAI-T	BIS-11
Caloric intake								
ρ	1	**0.438****	-0.030	0.137	-0.034	0.162	-0.064	0.081
p-value		**0.003**	0.846	0.364	0.812	0.247	0.651	0.560
n	57	44	44	46	52	53	53	54

BDI = Beck Depression Inventory; BEE = binge eating episode; BIS-11 = Barrat Impulsivity Scale; BITE = Bulimic Investigatory Test, Edinburgh (S = Symptoms subscale, G = Severity Subscale); BN = bulimia nervosa; STAI-S = State Anxiety Inventory; STAI-T = Trait Anxiety Inventory.

Supplementary Table S4 - Pearson's correlation coefficients and level of significance between caloric intake during BEE and levels of general and eating related psychopathology in BED

DIAGNOSIS	Caloric intake	BES	BDI	STAI-S	STAI-T	BIS-11
Caloric intake						
ρ	1	0.130	-0.004	-0.111	0.047	0.205
p-value		0.411	0.975	0.443	0.748	0.149
n	57	42	53	50	50	51

BDI = Beck Depression Inventory; BED = binge eating disorder; BEE = binge eating episode; BES = Binge Eating Scale; BIS-11 = Barrat Impulsivity Scale; STAI-S = State Anxiety Inventory; STAI-T = Trait Anxiety Inventory.
